# The effect of inverting decades and units on the retention of two-digit numbers in working memory: a matter of the output mode

**DOI:** 10.1007/s00426-024-02046-4

**Published:** 2024-11-11

**Authors:** Maisam Hayek, Avi Karni, Zohar Eviatar

**Affiliations:** 1 The Edmond J Safra Brain Research Centre for the Study of Learning Disabilities, University of Haifa, Haifa, Israel; 2 Department of Learning Disabilities, University of Haifa, Haifa, Israel; 3 Special Education Department, Sakhnin Academic College, Sakhnin, Israel; 4 The Sagol Department of Neurobiology, University of Haifa, Haifa, Israel; 5 Department of Psychology, University of Haifa, Haifa, Israel; 6 Institute for Information Processing and Decision Making, University of Haifa, Haifa, Israel

## Abstract

Number-word structure affects transcoding and relates to working memory capacity (WMc). We tested the relationship between the structure of number-words (inverted “Units-Decades (UD) format” or not “Decades-Units (DU) format”) and performance on the digit span task for recalling two-digit number-words presented verbally, using two modes of output. University students, native speakers of Arabic, who were also highly proficient in Hebrew, and native speakers of Hebrew, were asked to response by typing Arabic digits or recalling numbers verbally. Arabic and Hebrew speakers were equal in WMc for one-digit numbers in the two modes. However, when inversion was presented, performance was related to output and participants habits. In the typing response, both groups showed better performance for the DU format. In the native language (standard format), Arabic speakers (UD) remembered fewer numbers than Hebrew speakers (DU). Arabic speakers show inversion errors even in the noninverted format. For verbal responses, there were no differences between the groups in their standard format, and native speakers of Arabic weren’t affected by the format, while Hebrew speakers still preferred the DU format. Experience with inverted and noninverted language affected WMc but is also related to the output demands.

**Supplementary Information:**

The online version contains supplementary material available at 10.1007/s00426-024-02046-4.

## Introduction

Number-word systems all over the world are different in many aspects such as base and composition type (Comrie, 2005, 2006). Recent research has provided direct evidence for linguistic influences on numerical cognition, even in tasks that do not explicitly require verbal processing (Bahnmueller et al., 2019). In the present paper we did not test numerical cognition, rather, we are interested in the effects of number-word inversion on transcoding and its relation to working memory capacity (WMc). Previous research on our lab had suggested (Hayek et al., 2019, Hayek et al., 2025) that response mode is related to transcoding and in the present research we focused on the effect of response mode on WMc.

The inversion phenomenon is present in many languages such as Arabic, German, Dutch, Danish, Malagasy and Maltese as well as partly in Czech and Norwegian. It refers to the inverted order of the lexical elements in the syntactical organization of the names of numbers (Comrie, 2005, 2006). In these number-word systems, for example, the verbal number word 58 is referred to as “eight-and-fifty”, units before decades (UD), thus, number-words are inverted as compared to the standard written (“Arabic numerals”) notation, read from left to right (Zuber et al., 2009).

The inversion of units and decades of two-digit numbers can affect performance in tasks requiring calculation, with speed and accuracy reduced in comparison to languages in which there is no inversion (decades precede units, DU); that is, the numerical and verbal structures correspond (Helmreich et al., 2011; Göbel et al., 2014). In addition to the inversion effect on basic arithmetical abilities, inversion also affects the ability to transcode numbers (change notation from numerals to number-words and the reverse), reflecting the relationship between the numeral and the verbal system. In a series of recent studies in our lab (Hayek et al., 2019; Hayek et al., 2025) we tested native Arabic speakers (a language with number inversion) who are highly fluent bilinguals in Hebrew (a language without inversion). We examined the effects of inversion on transcoding written or spoken number names to digits by typing (Hayek et al., 2025) and handwriting (Hayek et al., 2019). Native speakers of Hebrew (exposed only to noninverted language- DU format) were tested as a control group. Both Arabic and Hebrew are written from right-to-left, but numbers are written from left-to-right. In these experiments, participants were presented with numbers either visually, (using either numerals (*Arabic* digits) or digit words), or auditorily, using digit words. The verbal target stimuli i.e., the multi-digit number-words, in both input modalities, were presented in one condition with decades first (twenty-four, DU) and in the other condition with units first (four and twenty, UD). The results showed that in Arabic, participants typed/wrote the numerals faster and more accurately when the number-words were presented in the DU format, regardless of whether the number-words were read or heard, even though this format is different from the one used in daily life. The two groups of Arabic and Hebrew speakers were equivalent in the typing/writing multi-digit numbers presented as *numerals*, but, when *number-words* were presented visually, a difference in performance was seen, in favor of the group with the noninverted number-word system (DU), i.e. in favor of the Hebrew speakers. We concluded that the slowness of transcoding standard Arabic number-words among native speakers of Arabic stems from the decades-units inversion, added to the difference in writing numbers and words (numbers are written from left to right in addition to the fact that words are written from right to left, which also occurs in Hebrew). We suggested that Arabic speakers are faster with DU format in Arabic (which is not standard or familiar), as a result of their proficiency in Hebrew, because the acquisition of a second language may bias the processing of numerical cognition (Prior et al., 2015; Kroll et al., 2015).

Studies on memory (e.g.; Baddeley et al., 1975; Naveh-Benjamin and Ayres 1986; Stigler et al., 1986) showed that long articulation times for digits makes more demands on the limited temporal resources of WMc; thus, the digit span in such a language is likely to be smaller compared to a language with a shorter articulation duration for digits. Variation in digit span was also found among bilinguals (e.g. Chincotta & Hoosain, 1995; da Pinto, 1991; Elliot, 1992; Ellis & Hennelly, 1980; Hoosain & Sallili, 1988). Bilinguals showed the effects of different factors: For example, *speech rate and differences in the articulatory complexity of digit names (word length)* between languages can influence the digit span (Baddeley, 1990). Among Welsh/ English bilinguals, faster reading time for English predicted larger digit spans for visually presented digits in English compared to Welsh, although the majority of subjects had rated themselves more proficient in Welsh (Ellis & Hennelly, 1980). In addition, *familiarity* (personal habits) also affects the digit span. For example, da Pinto (1991) suggested that a familiarity effect arising from massive practice for digits in the mother tongue may be responsible for the continued digit span superiority of first language over second language even under conditions of articulatory suppression. Thirdly, *practice in manipulating numbers* at home and in school also affects memory performance of bilinguals. For example, Naveh-Benjamin and Ayres (1986) suggested that among bilinguals, the language of teaching mathematics especially in elementary school, and intensive practice, may favor digit span tasks in that language, and override individual differences in number names between the first and second languages.

A recent meta-analysis by Grundy and Timmer (2017) provides compelling evidence for a bilingual advantage in WMc. This advantage was most pronounced in children, suggesting that the cognitive demands of early bilingual language acquisition may particularly enhance WMc development. WMc can also be related to number-word structure (Zuber et al., 2009). Numerals in the standard decimal system are defined by the value of single lexical elements (digits) and by the position of the digits within the number. Cognitive models of number transcoding posit that decoding numbers is affected by each of these two aspects independently of the other (e.g., McCloskey et al., 1985; McCloskey & Caramazza, 1987; Noel & Seron, 1995). This is indicated by the finding of two types of errors: syntactic errors (i.e., errors in the positional relation between the single elements such as writing 40076 instead of 467) and lexical errors (i.e., errors in designating the value of single digits such as writing 67 instead of 57). There is disagreement about whether transcoding between number formats passes along semantic (McCloskey, 1992) asemantic (Deloche & Seron, 1982a, b), or multiple routes (Cohen et al.,^1994^); the use of all these routes is dependent on numerical skills (Van Loosbroek et al., 2009).

It has been suggested (e.g., Barrouillet & Lepine, 2005; Camos, 2008; Zuber et al., 2009) that WMc affects transcoding ability not only through the ability to remember the lexical elements and their sequence, but also through the ability to manipulate the sequence of digits in multiple formats. Working memory measures are positively related to transcoding ability in both noninverting (UD) (e.g., English: Simmons et al., 2011) and inverting languages (DU) (e.g., Dutch: Imbo et al., 2014; German: Zuber et al., 2009). Differences are found in the types of errors made by children: In German and Dutch, nearly half of the errors were related to the inversion property (e.g., 29 was written as 92) whereas this type of error has not been observed in languages without the inversion property. Two recent studies on transcoding errors of two-digit numbers among Arab first graders (Ganayim et al., 2021) and Arabic-Hebrew bilingual university students (Ganayim et al., 2020) revealed errors related to the inversion format. The important role of executive working memory in both inverted and noninverted errors was reported (e.g., Pixner et al., 2011). Pixner et al. compared transcoding in the two number-word systems of Czech - one with and one without inversion – by testing first graders in a dictation task. The results showed that the children made less errors when asked to transcode number-words dictated in the noninverted (DU format) compared to the inverted format (UD). Here also, the errors were mostly related to the inversion phenomenon.

Some authors (e.g., Camos, 2008; Simmons et al., 2011; Zuber et al., 2009) have proposed that executive working memory is more important in children’s number transcoding in inverted number languages (e.g., German and Czech; Imbo et al., 2014; Zuber et al., 2009) than in noninverted number languages (e.g., English; Simmons et al., 2011). Transcoding number-words to numerals is presumed to require phonological representation in addition to symbolic visuospatial representation. When this process is more complex because of the requirement for inversion of the direction between units and decades, WMc may play a larger role in successful transcoding (Zuber et al., 2009). These studies highlight the potential role of WMc in processing numbers across different languages and number-word structures. WMc may be particularly relevant when dealing with inverted number-word formats, as they may require additional cognitive resources to process.

The studies mentioned above focused on the relation of memory components to transcoding ability in children. Building on these recent findings, our study aims to further elucidate the relationship between number-word structure (UD or DU), performance on the digit span task, a standard measure of WMc. Specifically, we tested Arabic and Hebrew speaking university students who were equivalent in WMc for 1-digits, but differed in whether their mother tongue included inversion in multi-digit numbers. It is important to emphasize that both Arabic and Hebrew script are written from right to left. Both uses Arabic digits as numerals, but only the Arabic language includes unit decades inversion. Thus, in Arabic, words are read from right-to-left, and numerals are also named from right to left (the number 23 is named as “three and twenty”). However, when multi-digit numbers are written in the digit format, children are taught to write numerals with the decades first (DU, left to right). Thus, a double inversion is needed in transcribing words to numerals: one, mechanically moving from right-to-left (in producing the verbal written form) but switching to left-to-right for writing numerals (as in Hebrew, for example) and, in addition, switching (as in German) from units-decades (verbal) to decades-units (numerals). Also, in typing multi-digit numbers at least one inversion is necessary; multi-digit numbers must be typed decades before units. Note that no such grounds for uncertainty are present in noninverting languages, e.g., Italian or English or Hebrew.

We compared the ability of Arabic and Hebrew participants on a multi-digit digit-span task. The list of numbers was presented verbally in a DU and a UD format in Arabic and in Hebrew. We evaluated the effect of reversing the order of decades and units in verbally (auditory) presented numbers on performance in two response conditions (modes): typing the numerals, and recalling the numbers verbally (orally). Typing requires, in addition to engaging WMc, the transcoding of the stimuli into a DU format, even when they are presented in the other format, UD. Such demands are not included in the verbal response. We hypothesized that when multi-digit numbers were presented in the UD format, there would be more demands on WMc compared to the DU format, and thus fewer numbers will be recalled, and that this would be more evident in the typing response mode than in the verbal mode.

All of our Arabic speaking participants are highly fluent in Hebrew. A subset of theses participants also tested in Hebrew. We hypothesize that when numbers are presented in a DU format, performance in L1 (Arabic) and L2 (Hebrew) will be equivalent, whereas when numbers are presented in UD format, performance will be better in L1 (which is an inverting language). We expected native Arabic speakers to show a smaller difference between the inverted UD and noninverted DU conditions than native Hebrew speakers because they have experience with both number formats.

## Method

### Participants

Forty-one university students, native speakers of Arabic (Arabic-Hebrew bilingual) (23 male, 18 female; mean age = 24.4 years, SD = 3.8) were tested and compared to thirty-four university students, native speakers of Hebrew (19 male, 15 female; mean age = 29.94 years, SD = 5.08) who were not familiar with a foreign language that uses UD inverted format in their number system. All participants were right-handed and neurological healthy (healthy, with normal hand motor abilities, normal hearing and normal or corrected vision).

Given that statistics of the speaker’s previous experiences and exposure to multi-digit number-words may lead to an efficient routine for retention and bias the results of the study, students of natural sciences, mathematics or economics, were not included.

All participants studied mathematics at school in their native language using Arabic digits. All of the participants passed the matriculation in Hebrew as well as a Hebrew and English language test as part of entrance test for the university. All the of native speakers of Arabic were highly proficient in Hebrew as a second language, as it is the majority language in their everyday life and the main (in many cases the only) language of teaching in the university. Native speakers of Hebrew were not familiar with a foreign language that uses inversion in their number system.

Participants were asked to confirm their consent to take part in the study after receiving an explanation of the experiment by signing the informed consent form. For their participation time in a research study, they received either credit points or monetary compensation (45 NIS). The study was approved by the Faculty of Education University of Haifa Human Experimentation Ethics Committee (Approval number: 298/15).

In order to compare the groups on general abilities, we tested them on computerized Corsi blocks (forward condition) and a task of completion of words in context and then recalling those words verbally (in the native language). In addition, they underwent the short version of the standard Raven’s Matrices. All participant underwent a test of typing numbers randomly from 1 to 20 to assess their speed of typing digits. There were no significant differences between the groups on any of these measures (*p* > .74).

### Stimuli, materials, and procedure

The stimuli were 1 and 2-digit numbers. 1-digit numbers were included to test whether the groups are comparable, and basically equal in memory abilities, when no inversion is present. The 2-digit numbers were selected randomly from the set between 21 and 98. Excluded from the stimulus set were the multiples of 10 (e.g., 20, 30, 40, …) and tie numbers (i.e., numbers wherein there was an identity in the digits denoting decades and units and no inversion is shown in the written format. (e.g. 33; 44). The complete task took 30–40 min.

All the participants performed the typing condition before the verbal condition. The test paradigm design was the following: for each response type (typing or verbal), participants first performed a set of four blocks of series of 1-digit numbers. There were 5 trials in each block. The first block contained a list of 2 numbers to be recalled in each trial. The second block contained a list of 3 numbers to be recalled in each trial. The third block contained a list of 4 numbers to be recalled in each trial and the fourth block contained a list of 5 numbers to be recalled in each trial. Overall, 70 1-digit numbers were presented for each type of response. The presentation rate of the numbers was one digit per second.

The participants performed another two different sets in each response type. Each set consist of 3 blocks of 2-digit numbers. The stimuli were presented in the DU format in one set and in the UD format for the other set. Each block also contained 5 trials. Overall, 45 2-digit numbers were presented for each set. The first block contained a list of 2 2-digit numbers to be recalled in each trial. The second block contained a list of 3 2-digit numbers to be recalled in each trial. The third block contained a list of 4 2-digit numbers to be recalled in each.

Since we were interested in the effect of cognitive habits, each group performed the blocks in the standard order of their native language first: Arabic speakers performed the UD blocks first and Hebrew speakers performed the DU blocks first. All participants performed the typing responses before the verbal responses, with a short break of 5 min between these conditions. Stimuli were not repeated in each response type. The series within the blocks were different between the condition and in each response type. All sets of numbers were presented verbally via earphones, approximately 1.5 s between each number in the series, spoken in a male voice.

The Arabic speaking participants who performed the typing conditions in both languages, always performed them in L1 first and after 10-minute break in L2.

The experiment was conducted individually in a quiet room via a computer and run using an E-prime script (2.0; Psychology Software Tools, Inc. Pittsburgh, PA). Participants were asked to respond by typing the numbers word as digits as quickly and accurately as possible, or orally, loudly and clearly, in the order of presentation.

Participants were instructed to press the space-bar of the keyboard (identical for both groups) for presenting the number series, a + symbol appeared on the screen simultaneously with the oral presentation of the number series, and disappeared with the end of each series, indicating that the response was required, at the end of which they pressed the ‘enter’ key.

Performance was assessed measuring *accuracy* –participants received a point if they recalled all the items in a sequence in the correct order.

We catalogued transcoding error types into errors related to the inversion property. Inverting the decade-unit order (e.g., 28 instead of 82). Errors that included other numbers such as 85 for 28 were defined as other errors.

## Results

### WMc for 1-digit numbers: Arabic and Hebrew speakers

**Fig. 1:**
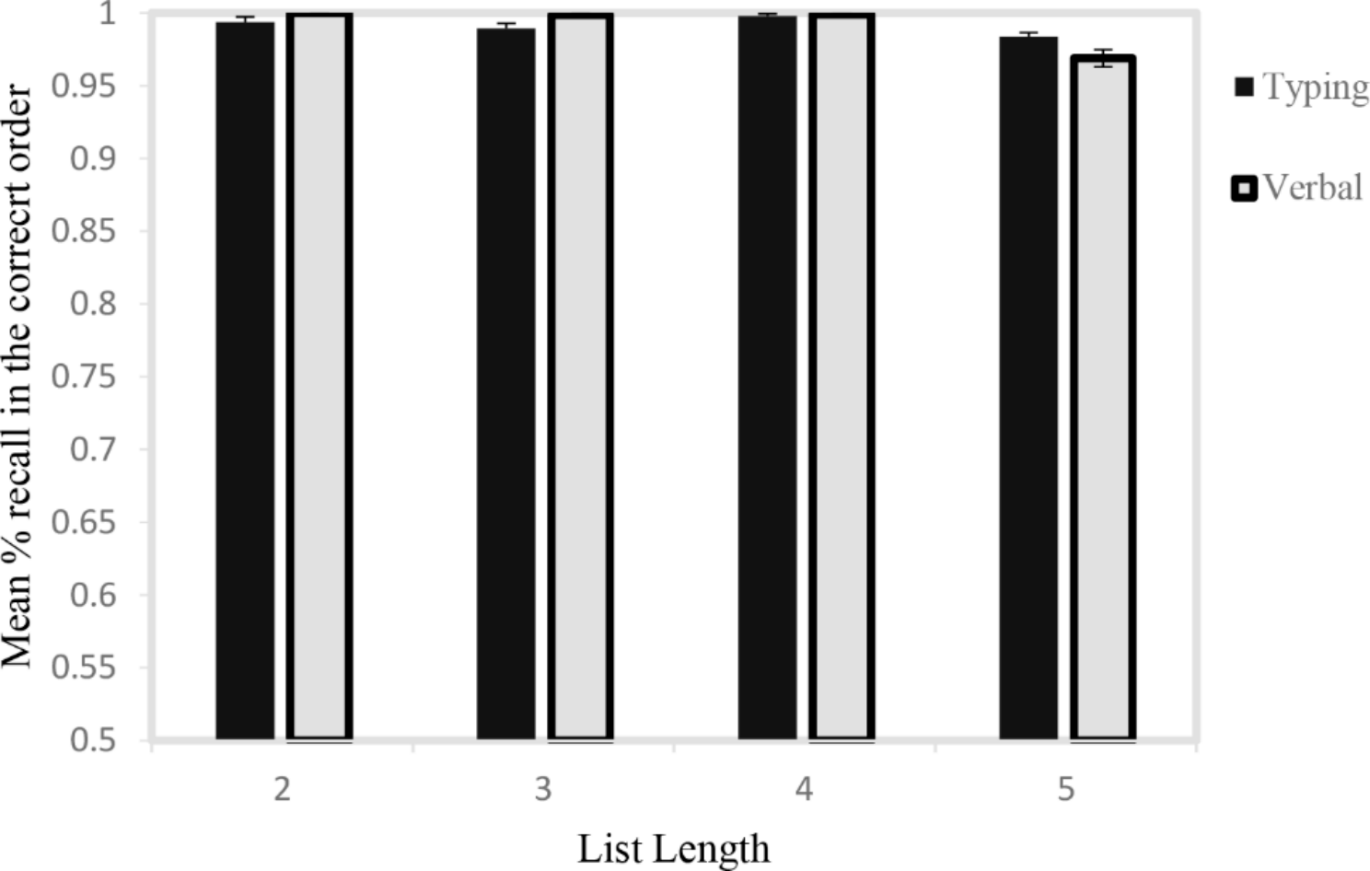
Percent recall in the correct order of 1- digit numbers for lists of different lengths. Error bars are standard errors

A 2 × 4 × 2 mixed GLM was run for Response Type (Typing, Verbal) and List Length (2,3,4,5) as within-subject factors, and Native Language (Arabic, Hebrew) as a between subjects factor. The analysis showed an interaction of Response by List Length (*F*(3,219) = 2.99, *p* = .03, *η*_*p*_^*2*^ = 0.01). Post hoc analyses revealed that the difference between typing and verbal responses approached significance in list lengths of 2 (*F*(1,74) = 3.08, *p* = .08, *η*_*p*_^*2*^ = 0.02), was significant in favor of verbal responses for list lengths of 3 (*F*(1,74) = 5.79, *p* = .019, *η*_*p*_^*2*^ = 0.04), and not significant for list of 4 (*F*(1,74) = 0.04, *p* = 1.0) and 5 (*F*(1,74) = 1.68, *p* = .198) numbers. These patterns are shown in Fig. 1. There was also a main effect of List Length, (*F*(3,219) = 40.07, *p* < .0001, *η*_*p*_^*2*^ = 0.19) reflecting that there were more errors in the list of 5 items than in the shorter lists. Importantly, there was no main effect of Native Language ( *F*(1,74) = 0.04, *p* = .83) and Native Language did not interact with List Length ( *F*(1,74) = 0.21, *p* = .88) or Response Type ( *F*(1,74) = 0.72, *p* = .38).

### Language structure and WMc

**Fig. 2:**
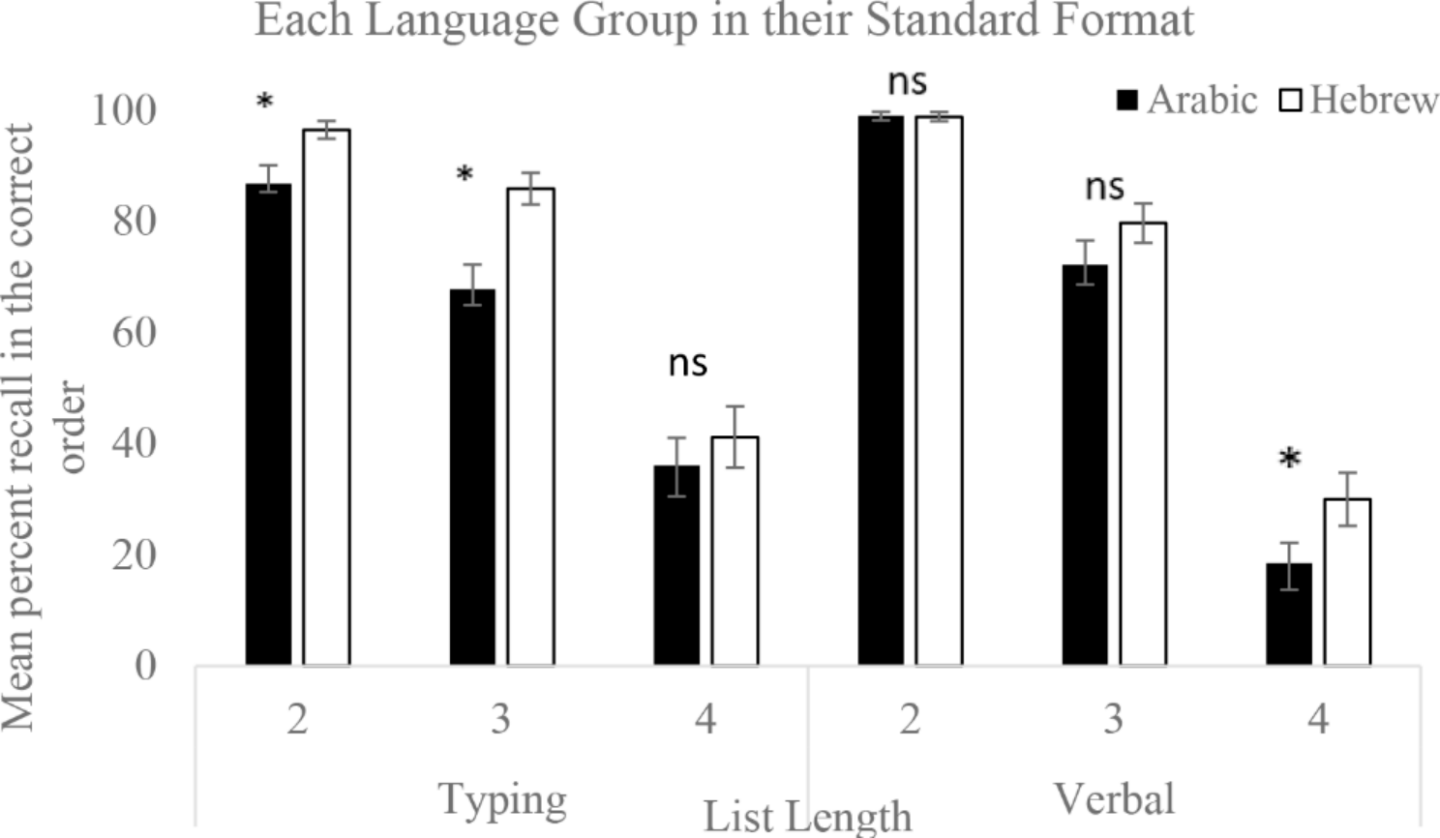
WMc for both Arabic and Hebrew native speakers, using varying sequence lengths in the standard format for each language (UD for Arabic; DU for Hebrew). Response was by typing or verbal. Significant effects of Format are indicated by *. Error bars are standard errors

To examine the effects of standard language structure for multi-digit numbers on WMc, we conducted a 2 × 3 × 2 mixed GLM with Native Language (Arabic, Hebrew) as a between-subjects factor, and Response Type (Typing, Verbal) and List Length (2,3,4) as within-subject factors. We compared the performance of the two groups in their standard format – UD for Arabic speakers and DU for Hebrew speakers. This analysis showed a trend towards a 3-way interaction between Native Language, List Length, and Response Type, *F*(2,146) = 2.73 *p* = .069, *η*_*p*_^*2*^ = 0.003. Planned comparisons showed that in the Verbal Response condition, the groups differed marginally only for List Lengths of 4, *F*(1,73) = 3.74, *p* = .057 *η*_*p*_^*2*^ = . 05. For the Typing response, the groups differed for list lengths of 2 (*F*(1,73) = 6.29, *p* = .014, *η*_*p*_^*2*^ = 0.08; and 3 items, *F*(1,73) = 10.78, *p* = .0016, *η*_*p*_^*2*^ = 0.13. The groups were equivalent for the List Length of 4. The differences between the groups for each Response Type for each List Length are shown in Fig. 2.

There was also an interaction between Response Type X List Length *F*(2,146) = 14.23, *p* = < 0.0001, *η*_*p*_^*2*^ = 0.16 and a main effect of List Length *F*(2,146) = 359.67, *p* < .0001 *η*_*p*_^*2*^ = 0.83; reflecting that there were more errors in the lists of 4 items than in the shorter lists. The main effect of Native Language *F*(1,73) = 6.4, *p* = .013 *η*_*p*_^*2*^ = 0.08, reflects better performance for Hebrew speakers than Arabic speakers.

### Effects of inversion on WMc for two-digit numbers

**Fig. 3:**
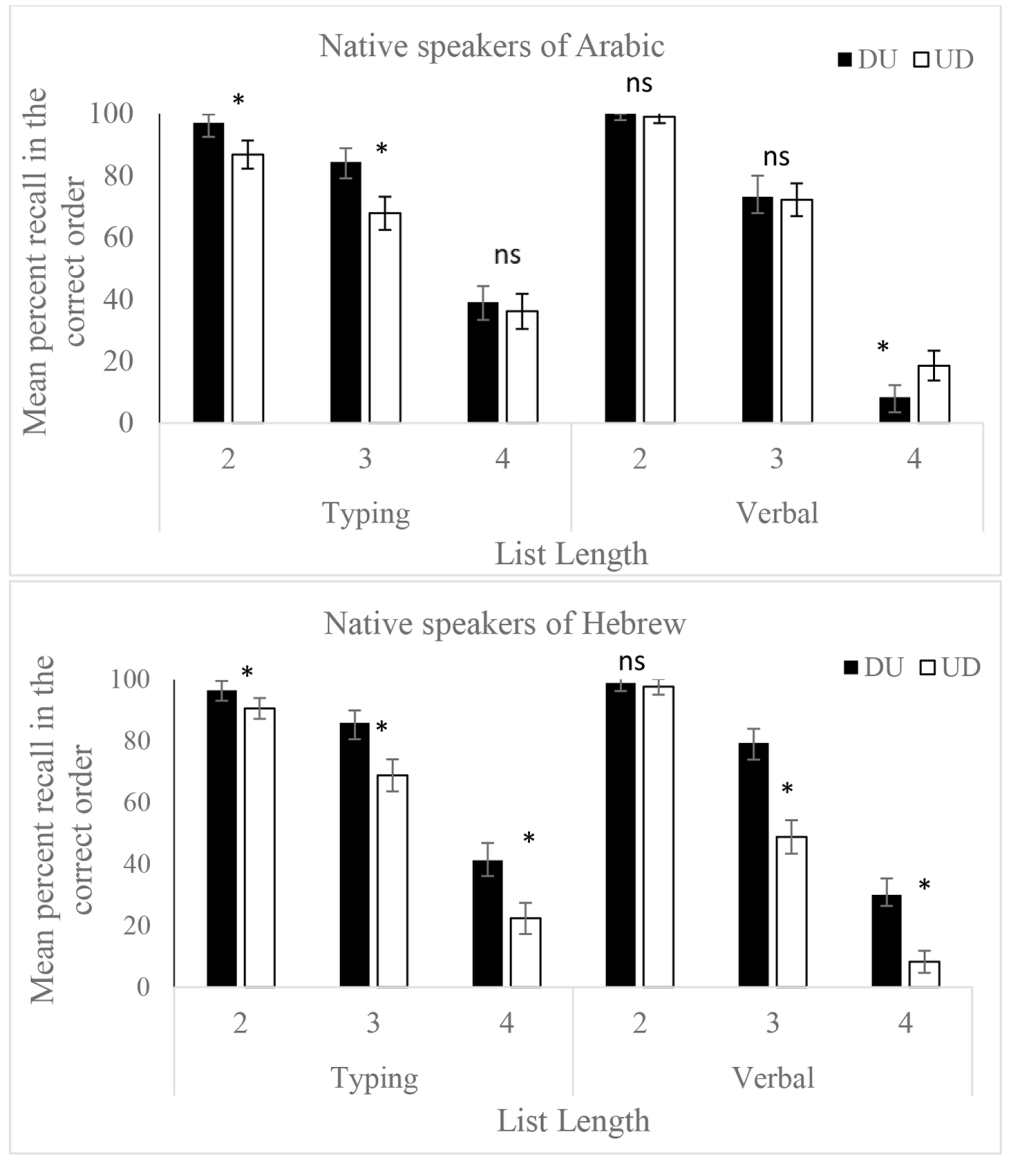
WMc for both Arabic and Hebrew native speakers, using varying sequence lengths, when stimuli were presented in inverted (UD) or noninverted (DU) format, and when response was by typing or verbal. Significant effects of Format are indicated by *. Error bars are standard errors

The manipulation of format was done for both groups in their native language, such that each group heard the numbers in the standard (UD for Arabic, DU for Hebrew) and the nonstandard (DU for Arabic, UD for Hebrew) format for their language. This allows an examination of inversion with each language group. A 2 × 3 × 2 × 2 mixed GLM was run on responses to 2-digit numbers, using Response Type (Typing, Verbal), List Length (2,3,4) and Format (UD, DU) as within subjects factor and Native Language (Arabic, Hebrew) as a between subjects factor. The 4-way interaction was not significant, *F*(2,73) = 2.16, *p* = .11. There were three significant 3-way interactions: specifically, Response X Format X Native Language (*F*(2,73) = 9.88 *p* = .002,* η*_*p*_^*2*^ = 0.003); Response X List Length X Native Language (*F*(2,146) = 5.6, *p* = .004,* η*_*p*_^*2*^ = 0.003) and Format X List Length X Native Language (*F*(2,146) = 9.58, *p* < .0001,* η*_*p*_^*2*^ = 0.005). The pattern shown in Fig. 3 indicates that both the Format and the Response Type, as well as the effect of changing the List Length (number of items in the set to be memorized) were differentially affecting performance in the 2 language groups.

To explore these interactions, GLM analyses were run separately for each Native Language group, using Format (UD, DU), List Length (2,3,4) and Response (Typing, Verbal) as within subject factors. The significant effects shown by each Native Language group are listed in Table 1. Patterns which differ between the groups are listed in bold.

**Table 1:** Main effects and interactions of Format, list length, and response mode in each native Language group

Effect	Arabic Speakers	Hebrew Speakers
Format X List Length X Response	Ns, *p* = .8	Ns, *p* = .08
**Format X Response**	***F*(1**,**80) = 8.68**, ***p*** **= .005**, ***η***_***p***_^***2***^ **= 0.007**	**Ns**, ***p*** **> .14**
Format X List Length	*F*(2,160) = 4.63, *p* = .01, *η*_*p*_^*2*^ = 0.005	*F*(2,132) = 13.36, *p* < .0001, *η*_*p*_^*2*^ = 0.01
List Length X Response	*F*(2,160) = 23.97, *p* < .0001, *η*_*p*_^*2*^ = 0.03	*F*(2,132) = 11.21, *p* < .0001, *η*_*p*_^*2*^ = 0.01
**Format**	**Ns**, ***p*** **= .14**	***F*(1**,**66) = 114.55**, ***p*** **< .0001**, ***η***_***p***_^***2***^ **= 0.05**
List Length	*F*(2,160) = 364.35, *p* < .0001, *η*_*p*_^*2*^ = 0.6	*F*(2,66) = 307.74, *p* < .0001, *η*_*p*_^*2*^ = 0.6
Response	*F*(1,180) = 10.31, *p* = .003, *η*_*p*_^*2*^ = 0.007	*F*(1,132) = 20.32, *p* < .0001, *η*_*p*_^*2*^ = 0.008

Significant differences are marked in bold

As shown in Fig. 3, Arabic speakers show different effects of Format when responding verbally or by typing. When responding verbally, list lengths of 2 and 3 showed no effects of inversion, and for series of 4 numbers, inversion had a significant *facilitative* effect (UD better than DU). Thus, when there were more digits and the task became harder, the familiar format (the standard inverted structure) resulted in better performance. When responding by typing, series of 2 and 3 numbers showed an advantage for noninverted stimuli (DU better than UD), and on lists with 4 items, format had no effect. This is reflected in the significant interaction between Format and Response Type for Arabic speakers. In contrast, Hebrew speakers showed the same pattern in both response modes, with Format affecting responses more strongly as List Length grows, and DU always better than UD.

#### Correlation of WMc in the different response types

Given that the mode of response seems to have a stronger effect among Arabic speakers than for Hebrew speakers, we explored the relationship between the two structures of number-words in each group for each response type in terms of WMc. The correlation coefficients are shown in Table 2. Note that responses were pooled over the different List Lengths.

**Table 2:** Correlations between performance in the UD and DU formats among arabic and hebrew speakers in the typing and the verbal response mode

	Typing (UD vs. DU)	Verbal (UD vs. DU)
Arabic speakers	*R* = .21, *p* > .19	*R* = .41, *p = .007
Hebrew speakers	*R* = .66, *p < .001	*R* = .63, *p < .0001

Overall, WMc performance with the two formats was highly correlated, except in the condition in which native speakers of Arabic had to type. Thus, in native speakers of Arabic, when responding by typing, WMc for number words in the noninverted format (DU condition) was uncorrelated with WMc for number words in the inverted format (UD condition). This supports the hypothesis that the effect of format for typing conditions for Arabic speakers is due to the requirements of typing, and not to the representation of the inverted number itself.

### Error analyses for Arabic and Hebrew speakers

**Fig. 4:**
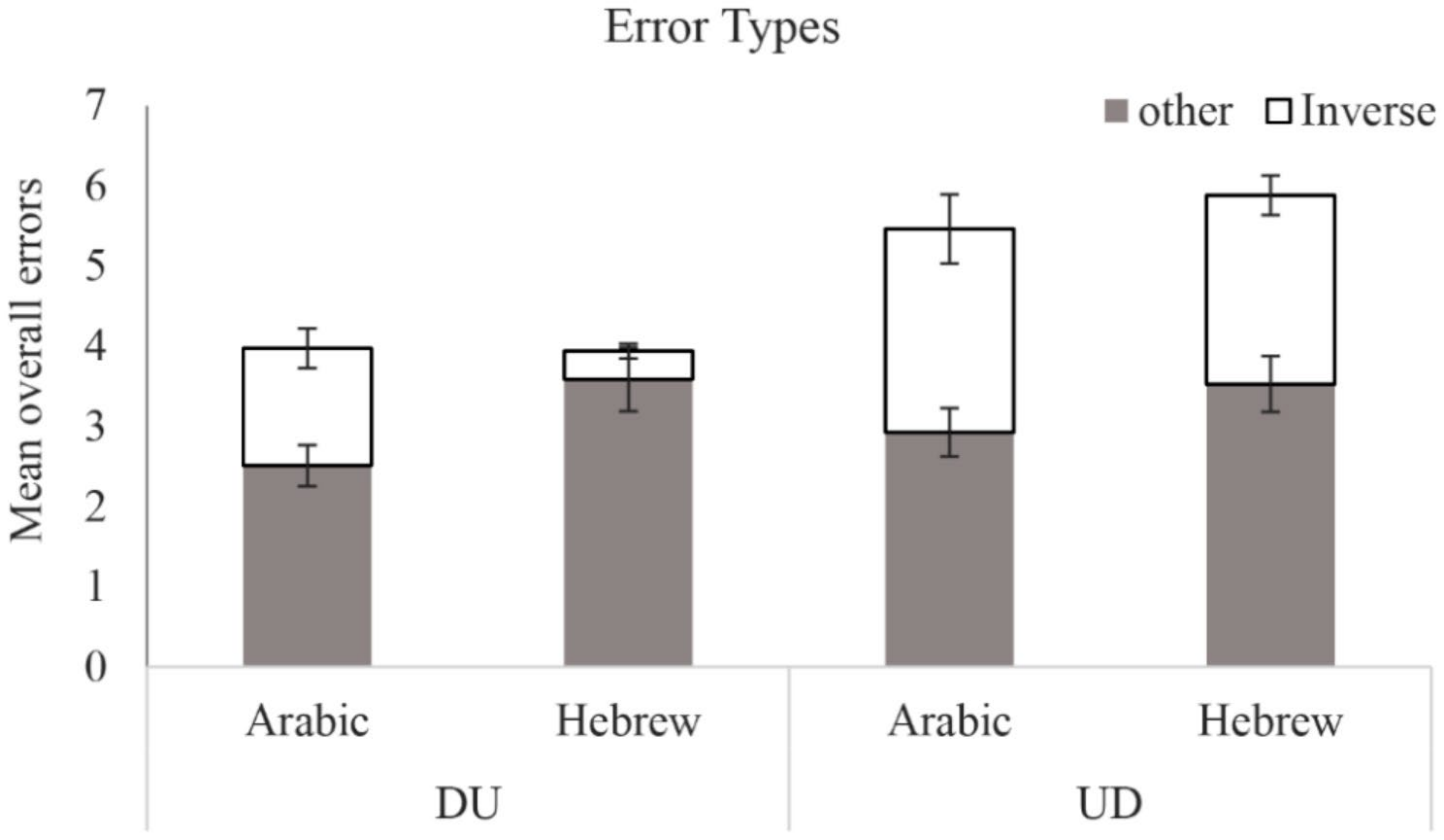
Mean of overall errors (inverted errors & other errors) for native speakers of Arabic and Hebrew, when stimuli were presented in inverted or noninverted format, and when response was by typing. Error bars are standard errors

There was no difference between the groups in the overall number of errors (*F*(1,73) = 0.16, *p* > .69). Recall that previous studies of children reported that in transcoding tasks, children who were tested in inverting languages made more inverting errors (e.g., Pixner et al., 2011; Zuber et al., 2009). In the current study, the protocol differed in two aspects: First, a typing response condition was included; second, both the inverted and noninverted formats of number words were presented to two groups of adults, one which is familiar with the inverted format (Arabic speakers) and one which is not (Hebrew speakers). The typing response mode necessitated inversion of the numerals in the UD format condition, allowing for inversion errors. This is because in typing participants need to enter decades before units in both formats. To test whether inversion errors are dependent on format or reflect the familiar language structure, errors from the typing condition were categorized into Inversion Errors (i.e., responses containing an inversion of the 2 digits comprising the target number (e.g., instead of 97 responding by 79), or any Other Errors. Overall participants made few errors, so the results were summed across List Length. These data are presented in Fig. 4. Analyses with List Length can be found in the supplementary materials and showed the same patterns.

A GLM was run for each error type separately as the dependent variable, with Format (UD, DU) as a within-subjects factor and Native language (Arabic, Hebrew) as a between-subjects factor. For Other Errors, there was a main effect of Native Language (*F*(1,73) = 5.46, *p* = .02,* η*_*p*_^*2*^ = 0.04), with Hebrew speakers making more errors of this type than Arabic speakers. There were no differences between the error rates in the two formats (*F*(1,73) = 0.39, *p* > .53) and no interaction (*F*(1,73) = 0.69, *p* > .3).

For Inversion Errors there was a main effect of Format (*F*(1,73) = 27.19, *p* < .0001,* η*_*p*_^*2*^ = 0.15), with more Inversion errors in the UD format than in the DU format, and also a main effect of Native Language (*F*(1,73) = 4.57, *p* = .04,* η*_*p*_^*2*^ = 0.03) with Arabic speakers making more Inversion errors than Hebrew speakers. Here also there was no significant interaction (*F*(1,73) = 2.48, *p* > .1).

There was a significant effect of Format for Inversion Errors, where both native language groups made significantly more inversion errors when the stimuli were presented in the inverted format than in the noninverted format, irrespective of language (for Arabic speakers: *t*(40) = 2.13, *p* = .03, *d* = 0.85; for Hebrew speakers: *t*(33) = 8.95, *p* < .001, *d* = 0.05). There was also a significant effect of familiarity of Language, with Arabic speakers committing more Inversion errors than Hebrew speakers, irrespective of Format.

### Arabic speakers in Arabic and Hebrew (response by typing)

Because the effects of inversion were only evident in the typing condition, 27 native speakers of Arabic were also tested in their L2 (Hebrew) in addition to Arabic, in the Typing Response condition. A 2 × 3 × 2 GLM analysis was run with Format (DU, UD), List Length (2,3,4) and Language (Arabic, Hebrew) as within subject factors. The dependent variable was the percent of errors. The analysis showed 3 significant 2-way interactions: as expected, there was an interaction between Format and List Length (*F*(2,52) = 5.57, *p* = .006,* η*_*p*_^*2*^ = 0.01), with format having stronger effects as the task becomes more difficult. The second two-way interaction was between Language X List Length (*F*(2,52) = 3.06, *p* = .05,* η*_*p*_^*2*^ = 0.005), where performance on length of 2 and 3 sequences was always better in L1, but there was no difference between L1 and L2 in the number of errors in sequences of 4 numbers. Importantly, the interaction between Format and Language *(F*(2,26) = 7.55, *p* = .01,* η*_*p*_^*2*^ = 0.01) was significant, reflecting that in the DU format, participants responded equivalently in the two languages, whereas in the UD format, they responded better in L1, Arabic, for which this is the standard format. There was a main effect of Language, with better performance in L1 than in L2 (*F*(1,26) = 5.26, *p* = .03,* η*_*p*_^*2*^ = 0.005). There was also a main effect of List Length, with more errors as the sequences of numbers were longer (*F*(1,52) = 166.78, *p* < .0001,* η*_*p*_^*2*^ = 0.42).

## Discussion

The main findings of the current study are that WMc, as measured by the digit span task, is dependent on a combination of factors: the format of the input to be recalled (number word structure DU vs. UD), the response mode, and one’s language habits. Importantly, in the standard version of the digit span task, in which single digits are verbally presented to be serially recalled, there were no differences between native speakers of Arabic and Hebrew, and no differences between the response mode conditions. However, when the response mode required inversion, performance was affected by input format and response mode: For verbal responses, Hebrew speakers performed better with the familiar noninverted (DU) format, while Arabic speakers showed no format preference for shorter sequences and a preference for their native inverted (UD) format in longer sequences. Arabic speakers, who are familiar with both UD and DU format due to their bilingualism, showed more flexibility in processing different number formats, particularly in verbal responses. In the typing response condition with 2-digit numbers, the effects of the standard number-word structure in the two languages, as well as the participants’ familiarity with these structures, were significant; both groups performed better with the noninverted (DU) format, regardless of their native language structure. Thus, response accuracy, specifically, the ability to recall the correct sequence of numbers, was contingent on whether the response was verbal or by typing, and was strongly affected by the familiarity of the participants with the process of the UD format, as necessitated by standard UD Arabic but not by standard DU Hebrew.

### Familiarity with different number-word structure

One of the novel aspects of the present research is that we compared ability to cope with series of numbers presented in two formats (DU, UD) in terms of WMc between participants, who were familiar with both inverted and noninverted structures due to their bilingualism, to those who are not. That is, we measured the ability of speakers of a noninverting language (Hebrew) to retain series of multi-digit numbers in a format with which they are unfamiliar. Moreover, we tested speakers of an inverting language (UD) (Arabic) who are nevertheless highly proficient speakers of the noninverting second language (DU), in both languages. We show that in the verbal response, Hebrew speakers always performed better when asked to recall series of multi-digit numbers in their familiar DU format than in the UD format. However, native speakers of Arabic weren’t affected by the format (UD or DU) in the smaller list length (2 & 3) and in fact, in the hardest list of 4 items, native speakers of Arabic showed a preference for their inverted native language (UD). For the harder list, participants still prefer to recall sequence of numbers verbally in their familiar inverted L1 (UD). We believe that this is because verbal recall does not require inversion as recall by typing. As can be seen, in the typing response, native speakers of Arabic and Hebrew showed the same pattern of performance in all list length: better performance for the DU than for the UD format.

**Fig. 5:**
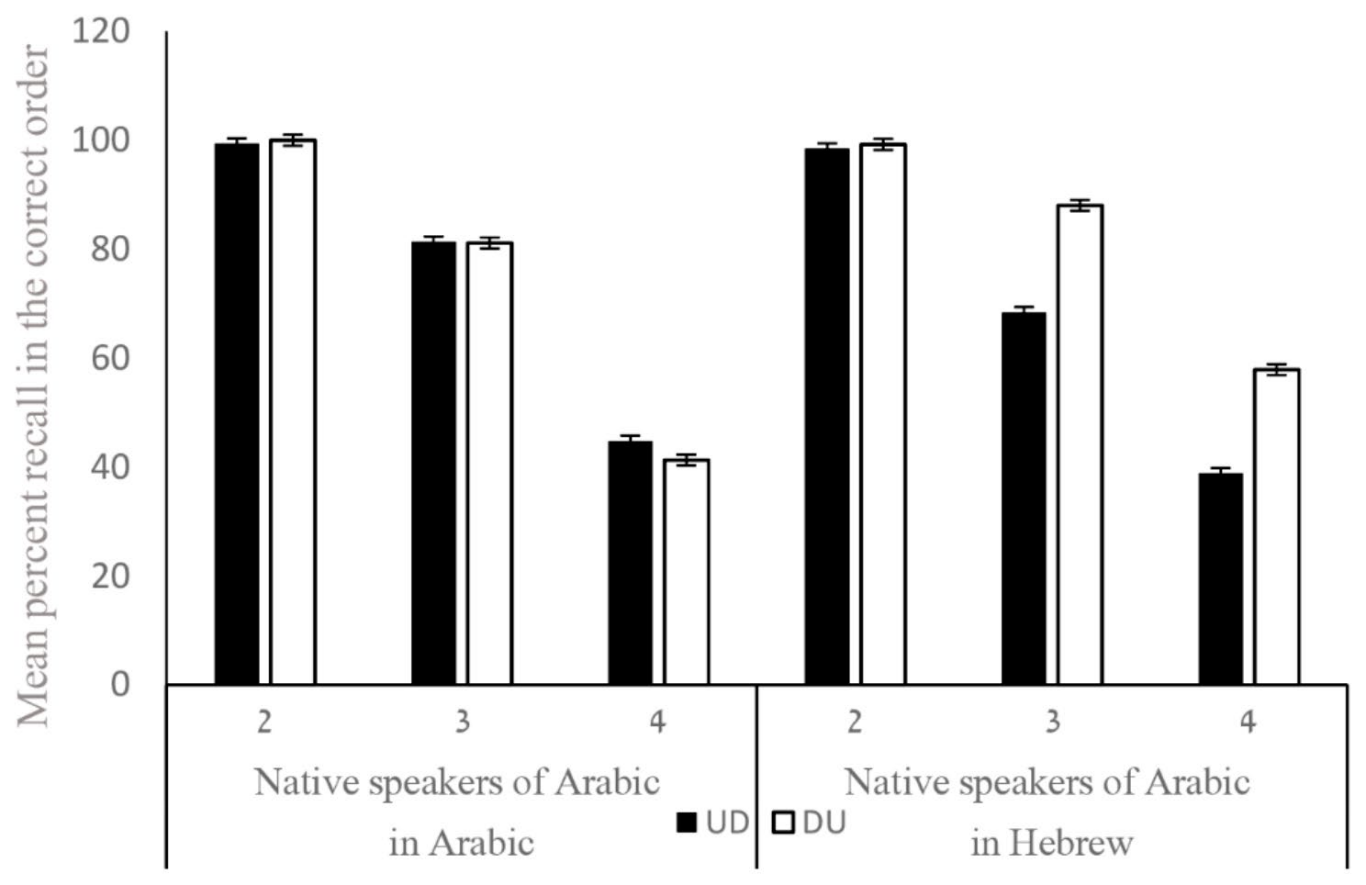
WMc for Native Speakers of Arabic in Arabic and Hebrew for sequences of different lengths, when stimuli were presented in the inverted and noninverted format and in the typing response mode. Error bars are standard errors

Our findings support the suggestion by Kroll and her colleagues (2015) that when bilinguals are highly proficient in their L2, it can even affect their L1 (in addition to the effects of L1 on L2). Specifically, when Arabic-Hebrew bilinguals were presented with numbers to be verbally recalled in Arabic, they performed equally well whether the numbers were presented in the UD or DU format (see Fig. 3). This pattern suggests that experience with a noninverting L2 (Hebrew) affected performance in L1 (Arabic). To further support this interpretation, we can refer to the results from the subset of Arabic speakers who were tested in both Arabic and Hebrew (see Fig. 5). These participants showed equivalent performance in the DU format across both languages in the typing condition, further indicating a transfer effect from L2 to L1.

Future research could investigate whether this effect is observed in Arabic speakers with varying levels of proficiency in noninverting languages, or in speakers of other inverting languages who acquire a noninverting L2. This would help determine the extent to which these findings can be generalized to other bilingual populations or if they are specific to Arabic-Hebrew bilinguals. The overall pattern of results suggests that exposure to both UD and DU number-word structures, as experienced by our Arabic-Hebrew bilingual participants, can lead to the development of flexible processing routines for both formats. This flexibility was particularly evident in the verbal response condition, where Arabic speakers showed equivalent performance for both UD and DU formats. Thus, the current results suggest that the level of familiarity with different number-word structures influences WMc for number sequences in specific formats (Grundy and Timmer 2017). However, this influence is moderated by the response mode (verbal or typing). In the verbal response condition, familiarity with both formats led to equivalent performance across formats for Arabic speakers. In contrast, in the typing condition, both Arabic and Hebrew speakers showed better performance with the DU format, suggesting that the demands of the response mode can override the effects of linguistic habits. These findings highlight the complex interplay between linguistic experience, number format, and response modality in determining WMc for number sequences. They underscore the importance of considering both language background and task demands when assessing numerical cognitive processes (Schliephake et al., 2023).

### Language habits affect WMc

The use of the typing response, together with the inclusion of a noninverting condition (DU) for speakers of an inverting language (UD), allowed us to uncover an unexpected phenomenon: native speakers of Arabic still made inversion errors when presented with numbers in a format that does not require inversion, the DU format (see Fig. 4). We interpret this as an indication that young adult native speakers of Arabic may employ an automatic inversion routine. There were no significant differences in the overall error rates in speakers of Arabic and their Hebrew speaking peers and both groups showed more inversion errors when the numbers to be recalled in the inverted UD format. However, native speakers of Hebrew made a significantly higher number of noninversion errors (syntactic and lexical), while native speakers of Arabic made a significantly higher number of inversion errors. These findings are in line with the observation in children, where the native language number format was a factor in determining the number of inversion errors in a number of tasks (e.g., Pixner et al., 2011).

### Response mode affects WMc

The current results also show that the format of the number-words had a stronger effect in the typing condition than in the verbal condition. It may be the case, that the typing of multi-digit numbers presented in the UD format necessitates at least one inversion of units and decades: in the mental representation of the target number and/or in the typing direction (as multi-digit numbers must be typed decades before units). Verbal responses do not necessitate such inversions, as responses are free to be inverted or not.

In the verbal condition, only Hebrew speakers showed the effect of format, with better performance in the standard DU format than the nonstandard unfamiliar UD format. Arabic speakers show no effect of format in the verbal condition. They performed equivalently in the standard UD format and in the nonstandard DU format. The UD format is not familiar to Hebrew speakers, and thus limited their WMc for these series of numbers as compared to the noninverted DU format. Performance of the Arabic speakers in the verbal response condition reflected the ability of native speakers of Arabic to deal with both UD and DU format, as a result of their high proficiency in a noninverting second language. It seems that increasing experience with the UD format may result in direct memory retrieval of the stipulated numbers and thus decrease working memory load (Barrouillet & Lepine, 2005; Daubert & Ramani 2019; Zuber et al., 2009).

A difference in performance that is contingent on response mode was also found by Brysbaert et al. (1998). Using an addition task, they found differences between Dutch (a language with inversion) and French (a noninverting language) speaking participants when response was oral, but not when response was done by typing. They concluded that language characteristics (such as number inversion) do not affect mathematical processes. Our results show the opposite pattern: the groups respond quite similarly in the oral response condition when no inversion was needed, but differently in the typing condition. The divergence in findings is probably due to different task demands, our study measured WMc and did not require mathematical computation. Our results have implications for the representation of numbers in working memory. The finding supports the notion that representation of numbers in working memory **is not** affected by the format but only by the response mode of the task. When the response mode required inversion in order to type correctly (as for UD format) both Hebrew speakers (for whom this format is novel) and Arabic speakers (for whom this format is standard) made more errors than when the response mode did not require an inversion. As shown in Table 2, the only condition in which there is no significant correlation between performance in the two formats, is in the typing condition, for speakers of Arabic, suggesting that output demands affected their WMc in the two format conditions.

Another finding that highlights the critical role of response mode is related to the comparison between Arabic and Hebrew speakers in their standard formats (UD and DU respectively). While both groups performed similarly in verbal recall, significant disparities emerged in the typing condition. Hebrew speakers outperformed Arabic speakers when typing, particularly for shorter sequences. This disparity highlights how the demands of typing interact with linguistic habits and number-word structure, suggesting that the inversion required in Arabic may impose additional cognitive load during transcoding. These findings underscore that the effects of number-word structure on WMc are not uniform, but are strongly modulated by task-specific output requirements.

In conclusion, the findings of this study make several important contributions to our understanding of WMc. The results indicate that performance of the recall task was affected by familiarity with the number format and the habits of the participants with inversion. These effects were modulated by the different response forms. Secondly, our results provide novel insights into how bilingualism affects WMc for numbers, showing that participants habits and experience with different number-word structures can lead to flexible mental representations.

## Electronic supplementary material

Below is the link to the electronic supplementary material.

Supplementary Material 1

## Data Availability

No datasets were generated or analysed during the current study.

## References

[CR1] Baddeley, A. D. (1990). *Human memory: Theory and practice*. Allyn and Bacon.

[CR2] Baddeley, A. D., Thomson, N., & Buchanan, M. (1975). Word length and the structure of short-term memory. *Journal of Verbal L Earning and Verbal Behavior*, *14*, 575–589.

[CR3] Bahnmueller, J., Maier, C. A., Göbel, S. M., & Moeller, K. (2019). Direct evidence for linguistic influences in two-digit number processing. *Journal of Experimental Psychology: Learning Memory and Cognition*, *45*(6), 1142. 30024256 10.1037/xlm0000642

[CR4] Barrouillet, P., & Lepine, R. (2005). Working memory and children’s use of retrieval to solve addition problems. *Journal of Experimental Child Psychology*, *91*, 183–204. 15925643 10.1016/j.jecp.2005.03.002

[CR5] Brysbaert, M., Fias, W., & Noel, M. P. (1998). The whorfian hypothesis and numerical cognition: Is ‘‘twenty four processed in the same way as ‘‘four and twenty? *Cognition*, *66*, 51–77. 9675978 10.1016/s0010-0277(98)00006-7

[CR6] Camos, V. (2008). Low working memory capacity impedes both efficiency and learning of number transcoding in children. *Journal of Experimental Child Psychology*, *99*, 37–57. 17854821 10.1016/j.jecp.2007.06.006

[CR7] Chincotta, D., & Hoosain, R. (1995). Reading rate, articulatory suppression and bilingual digit span. *European Journal of Cognitive Psychology*, *7*, 201–211.

[CR8] Cohen, L., Dehaene, S., & Verstichel, P. (1994). Number words and number non-words. A case of deep dyslexia extending to Arabic numerals. *Brain*, *117*, 267–279. 8186954 10.1093/brain/117.2.267

[CR9] Comrie, B. (2005). Endangered numeral systems. In J. Wohlgemuth, & T. Dirksmeyer (Eds.), *Bedrohte Vielfalt: Aspekte Des Sprach(en)tods [Endangered diversity: Aspects of language death]* (pp. 203–230). Weissensee.

[CR10] Comrie, B. (2006). *Numbers, language, and culture*. Paper presented at the Jyvaskyla Summer School, Jyvaskyla, Finland.

[CR11] da Pinto, C., A (1991). Reading rates and digit span in bilinguals: The superiority of mother tongue. *International Journal of Psychology*, *26*, 471–483.

[CR12] Daubert, E. N., & Ramani, G. B. (2019). Math and memory in bilingual preschoolers: The relations between bilingualism, working memory, and numerical knowledge. *Journal of Cognition and Development*, *20*(3), 314–333.

[CR13] Deloche, G., & Seron, X. (1982a). From one to 1: An analysis of a transcoding process by means of neuropsychological data. *Cognition*, *12*, 119–149. 6890429 10.1016/0010-0277(82)90009-9

[CR14] Deloche, G., & Seron, X. (1982b). From three to 3: A differential analysis of skills in transcoding quantities between patients with Broc’s and Wernicke’s aphasia. *Brain*, *105*, 719–733. 7139252 10.1093/brain/105.4.719

[CR15] Elliot, J. M. (1992). Forward digit span and articulation speed for malay, English, and two Chinese dialects. *Perceptual and Motor Skills*, *74*, 291–295.

[CR16] Ellis, N. C., & Hennelly, R. A. (1980). A bilingual word-length effect: Implications for intelligence testing and the relative ease of mental calculation in Welsh and English. *British Journal of Psychology*, *50*, 449–458.

[CR17] Ganayim, D., Ganayim, S., Dowker, A., & Olkun, S. (2020). Linguistic effects on the processing of two-digit numbers. *Open Journal of Modern Linguistics*, *10*.

[CR18] Ganayim, D., Ganayim, S., Dowker, A., & Olkun, S. (2021). Transcoding errors of two- digit numbers from arabic digits into verbal numbers and from verbal numbers into arabic digits by Arab First Graders. *Journal of Cognitive Education & Psychology*, *20*(2).

[CR20] Göbel, S. M., Moeller, K., Pixner, S., Kaufmann, L., & Nuerk, H. C. (2014). Language affects symbolic arithmetic in children: The case of number word inversion. *Journal of Experimental Child Psychology*, *119*, 17–25. 24269580 10.1016/j.jecp.2013.10.001

[CR19] Grundy, J. G., & Timmer, K. (2017). Bilingualism and working memory capacity: A comprehensive meta-analysis. *Second Language Research*, *33*(3), 325–340.

[CR21] Hayek, M., Karni, A., & Eviatar, Z. (2019). Transcoding number words by bilingual speakers of Arabic: Writing multi-digit numbers in a units-decades inverting language. *Writing Systems Research*. 10.1080/17586801.2020.1787298

[CR1000] Hayek M, Dorfberger S, Eviatar Z, & Karni A (2025). Transcoding number words to typed multi-digit numerals: revisiting the strange case in Arabic bilinguals. *Psychological Research*, *89*, 70. 10.1007/s00426-025-02097-1 40082282 10.1007/s00426-025-02097-1 PMC11906526

[CR22] Helmreich, I., Zuber, J., Pixner, S., Kaufmann, L., Nuerk, H. C., & Moeller, K. (2011). Language effects on children’s non-verbal number line estimations. *Journal of Cross-Cultural Psychology*, *42*(4), 598–613.

[CR23] Hoosain, R., & Sallili, F. (1988). Language differences, working memory, and mathematical ability. In M. M. Gruneberg, P. E. Morris & R. N. Sykes (Eds.), *Practical Aspects of Memory: Current Research and Issues*, Vol. 2: *Clinical and Educational Implications*, pp. 512–517. Chichester: John Wiley & Sons.

[CR24] Imbo, I., Bulcke, C. V., Brauwer, J. D., & Fias, W. (2014). Sixty-four or four-and-sixty? The influence of language and working memory on children’s number transcoding. *Frontiers in Psychology*. 10.3389/fpsyg.2014.00313 24782811 10.3389/fpsyg.2014.00313 PMC3990049

[CR25] Kroll, J. F., Dussias, P., Bice, E., K., & Perrotti, L. (2015). Bilingualism, Mind, and Brain. *Annual Review of Linguistics*, *1*, 377–394. 28642932 10.1146/annurev-linguist-030514-124937 PMC5478196

[CR26] McCloskey, M. (1992). Cognitive mechanisms in numerical processing: Evidence from acquired dyscalculia. *Cognition*, *44*, 107–157. 1511584 10.1016/0010-0277(92)90052-j

[CR28] McCloskey, M., & Caramazza, A. (1987). Cognitive mechanisms in normal and impaired number processing. In G. Deloche, & X. Seron (Eds.), *Mathematical disabilities* (pp. 201–219). Erlbaum.

[CR27] McCloskey, M., Caramazza, A., & Basili, A. G. (1985). Cognitive mechanisms in number processing and calculation: Evidence from dyscalculia. *Brain and Cognition*, *4*, 171–196. 2409994 10.1016/0278-2626(85)90069-7

[CR29] Naveh-Benjamin, M., & Ayres, T. J. (1986). Digit span, reading rate, and linguistic relativity. *Quarterly Journal of Experimental Psychology*, *38A*, 739–751. 10.1080/14640748608401623 3809578

[CR30] Noel, M. P., & Seron, X. (1995). Lexicalization errors in writing arabic numerals. *Brain and Cognition*, *29*, 151–179. 8573330 10.1006/brcg.1995.1274

[CR32] Pixner, S., Zuber, J., Hermanova, V., Kaufmann, L., Nuerk, H. C., & Moeller, K. (2011). One language, two number-word systems and many problems: Numerical cognition in the Czech language. *Research in Developmental Disabilities*, *32*, 2683–2689. 21763104 10.1016/j.ridd.2011.06.004

[CR33] Prior, A., Kats, M., Mahajina, I., & Rubinsten, O. (2015). Number word structure in first and second language influences arithmetic skills. *Frontiers in Psychology*, *6*, 266. 25852591 10.3389/fpsyg.2015.00266 PMC4362083

[CR35] Schliephake, A., Bahnmueller, J., Willmes, K., Koch, I., & Moeller, K. (2023). Influences of cognitive control on number processing: New evidence from switching between two numerical tasks. *Quarterly Journal of Experimental Psychology*, *76*(11), 2514–2523. 10.1177/17470218231154155 PMC10585943 36655942

[CR34] Simmons, F. R., Willis, C., & Adams, A. M. (2011). Different components of working memory have different relationships with different mathematical skills. *Journal of Experimental Child Psychology*, *111*, 139–155. 10.1016/j.jecp.2011.08.011 22018889 10.1016/j.jecp.2011.08.011

[CR36] Stigler, J. W., Lee, S., & Stevenson, H. W. (1986). Digit memory in Chinese and English: Evidence for a limited store. *Cognition*, *23*, 1–20. 3742988 10.1016/0010-0277(86)90051-x

[CR37] Van Loosbroek, E., Dirkx, G. S. M. A., Hulstijn, W., & Janssen, F. (2009). When the number line involves a delay: The writing of numbers by children of different arithmetical abilities. *Journal of Experimental Child Psychology*, *102*, 26–39. 10.1016/j.jecp.2008.07.003 18782636 10.1016/j.jecp.2008.07.003

[CR38] Zuber, J., Pixner, S., Moeller, K., & Nuerk, H. C. (2009). On the language specificity of basic number processing: Transcoding in a language with inversion and its relation to working memory capacity. *Journal of Experimental Child Psychology*, *102*, 60–77. 10.1016/j.jecp.2008.04.003 18499120

