# Supplementary material for: The effect of inverting decades and units on the retention of two-digit numbers in working memory: a matter of the output mode
Source: Psychol Res. 2024 Nov 11;89(1):2. doi: 10.1007/s00426-024-02046-4 (PMC11554729; doi:10.1007/s00426-024-02046-4)
Supplement: Supplementary file 1 — Supplementary Material 1 [file 426_2024_2046_MOESM1_ESM.docx]

**Supplementary**

**Error analysis for Arabic and Hebrew speakers:**

There was no difference between the groups in the overall number of errors (p>0.69). Recall that previous studies of children reported that in transcoding tasks, children who were tested in inverting languages made more inverting errors (e.g., Pixner et al.,[2011](http://www.ncbi.nlm.nih.gov/pmc/articles/PMC3733006/#B29); Zuber et al.,2009)). In our study, the typing response condition required the transcoding of verbal number names to numerals. In order to examine the effects of Format on these responses, errors were categorized into Inversion Errors (i.e., responses containing an inversion of the 2 digits comprising the target number (e.g., instead of 97 responding by 79), or any Other Errors.

A GLM was run for each error type separately as the dependent variable, with Format (UD, DU) and List Length (2,3,4) as a within-subjects factors and Native language (Arabic, Hebrew) as a between-subjects factor. The pattern of results is shown in Figure 6. For Inversion Errors there was a three-way interaction between these factors, *F*(2,146)=12.25, *p*<.000, *η_p_^2^*=.14. As shown at the top panel of figure 6, there were no differences between the two groups in the UD format and list lengths of 3 and 4 in the amount of inversion errors. While at the DU format native speakers of Arabic made significantly more inversion errors than Hebrew speakers in list lengths of 3 and 4. Furthermore, at list length of 4, native speakers of Arabic made more inversion errors at the DU format, while at the UD format, more inversion errors were made among native speakers of Hebrew. There was also a main effect of Format (*F*(2,146)=27.19, *p<.0001, η_p_^2^*=.27), with more Inversion errors in the UD than in the DU condition; a main of List Length, *F*(2,146)=67.06, *p*<.0001, *η_p_^2^*=.48, with the number of Inverse errors growing with List Length; and a main effect of Native Language, *F*(1,73)=4.57, *p*=.03, *η_p_^2^*=.06 with Arabic speakers making more Inversion errors than Hebrew speakers.

|  |
| --- |
|  |

Figure 6: Mean of overall errors (inverted errors & other errors) for native speakers of Arabic and Hebrew, when stimuli were presented in inverted or noninverted format, and when response was by typing.

For Other Errors, there was only a main effect of List Length, *F*(2,146)=210, *p*<.001, *η_p_^2^*= .74; and a main effect of Native Language *F*(1,73)=5.46, *p*=.02, *η_p_^2^*=.07. It can be seen that Arabic speakers made more Inverse errors and Hebrew speakers made more Other errors.
